# Physiological Correlates of Processing Health-Related Information: An Idea for the Adoption of a Foreign Field

**DOI:** 10.3390/nursrep11010017

**Published:** 2021-03-17

**Authors:** Cornelia Geukes, Horst M. Müller

**Affiliations:** 1 Centre for ePublic Health Research, University of Bielefeld, 33615 Bielefeld, Germany; cornelia.geukes@uni-bielefeld.de; 2 Experimental Neurolinguistics Group, Department of Linguistics, University of Bielefeld, 33615 Bielefeld, Germany

**Keywords:** neurophysiology, health literacy, information processing, health related information, physiological measurement

## Abstract

Measuring health may refer to the measurement of general health status through measures of physical function, pain, social health, psychological aspects, and specific disease. Almost no evidence is available on the possible interaction of physiological measures and correlating emotional–affective states that are triggered by dealing with individual health-relevant issues and their specific processing modes. Public health research has long been concerned with the processing of health-related information. However, it is not yet clear which factors influence access and the handling of health-related information in detail. One way to close this research gap could be adopting methods from neurocognitive experiments to add psychophysiological data to existing approaches in health-related research. In this article, we present some of these methods and give a narrative overview and description of their usefulness for enlarged research in public health.

## 1. Introduction

Frequently, there is a lack of knowledge about learning conditions and information conditions of individuals when it comes to health-related information. It seems that it is not sufficient to make information simple and user-friendly. Many individuals need support in understanding, evaluating, and using health-related information [1]. According to earlier studies, information design should be adapted to be suitable to information requirements [2,3,4]. However, there is a lack of knowledge at several levels concerning information requirements. This is due to two different reasons. First, at the level of information presentation, there is a lack of data to prove which modality is the most effective for understanding health-related information under different circumstances. Evaluation of interventions with large samples focusing on visual as well as auditory modalities are missing such that no overarching conclusions can be drawn. Second, this is a research gap urgently needing to be closed at the level of cognitive information processing. In other words, there is a lack of knowledge about the impact and the function of health-related information on the cognitive level and about the underlying process which is used to make a health-related decision.

Recent public health studies focused on the impact and the relationship between health-related information and health outcomes [5]. The concept of health literacy is exactly concerned with this relationship and means the access, the appraising, and the application of health-related information [6]. These human skills affect health-related decisions and are influenced by personal determinants such as age, gender, and education. These complex relationships can be influenced and changed within a lifespan. Accordingly, health literacy affects not only individual health but also quality of life and empowerment [6]. Since the 1990s, large-scale surveys [1,7,8,9] have been carried out that have evaluated health competence of the population using various survey instruments. These have enormously fueled the discussion about health literacy because it has become apparent that more than half the population (Germany, other European countries, and USA) has a low health literacy level such that health-related decisions are hardly possible in an informed, self-determined way [8,10]. Results show that the population does not or does not sufficiently understand health-related information, and that people hardly discuss it with peers in appropriate structures. Low health literacy is associated with poor health outcomes. More research is needed to understand the mechanisms and pathways of its effects [11,12]. In order to improve health outcomes and individual health literacy, information has been simplified mostly via the text of written materials. For the last two decades, intervention studies have been designed to improve the comprehensibility of health-related information [4,13]. However, the interventions have had varying degrees of success. This is reflected, for example, in the fact that the health literacy level measured in individuals has not been able to be increased significantly over the last two decades [1]. However, a generally applicable suitable solution has not yet been found. This could be due to the fact that public health research makes use of empirical methods following the social sciences, and the questions that can be answered with social sciences have apparently not desirably contributed to answering the question of bias in the processing and communication of health information.

So far, behavior-based methods have been used to prove whether the individual perceives, processes, and stores relevant content. These methods could be interviews to query knowledge in a dialogue or rating studies with questionnaires [14,15] applied at different points in time (immediate, after 1 week, after 3 months). There is now a chance for public health to make use of further interdisciplinary requirements and efforts [16]. Other disciplines provide methods that can allow asking questions differently and receiving different answers accordingly. Disciplines concerned with human health use numerous findings to control or improve the use and handling of health-related information. This should lead to two findings: 1. Expect findings with regard to the adequate processing of health-related information so that it is understood as well as possible; 2. People should be strengthened in their application of this information and their competencies should be increased. However, concepts such as health literacy show repeatedly that not all efforts have brought the desired results so far. The competence of the population in dealing with health information is not reaching a satisfactory level.

## 2. The Cognitive Processing of Health-Related Information and Its Measurement

**Figure 1:**
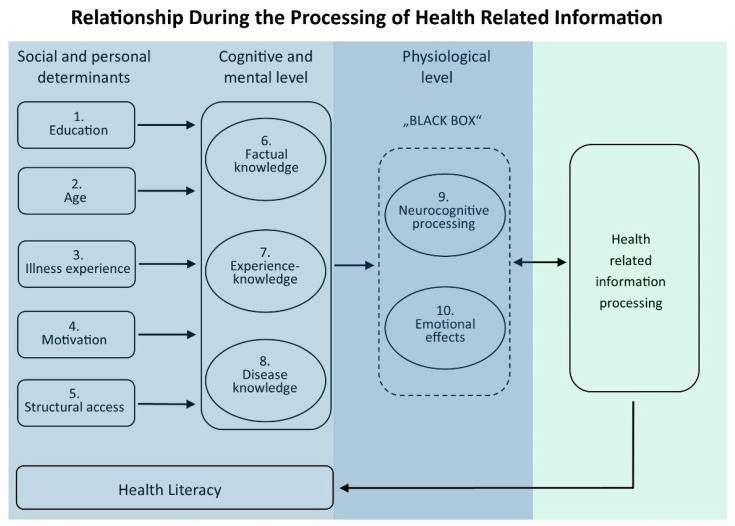
Block diagram showing major aspects influencing processing of health-related information.

To date, there are only a few results in terms of understanding and using modified health-related information. The relationship between health information and its understanding currently appears to be a “Black Box” (see Figure 1). Neuroscientific methods could contribute to an approach to this problem. Different verifiable emotional reactions to certain health information could lead to the information being processed differently from other factual/concrete information. The question that remains is the fundamental causal relationship between the presentation of health-related information and its understanding and further application. What is in between, and what appears to be a “Black Box”, is the cognitive processing of health-related information.

Social and personal determinants can influence the cognitive and mental level of an individual (see Figure 1, 1.–9.). Education (1.) lays a foundation for the knowledge of what an individual acquires in the course of their life. Age (2.) is also a factor because knowledge is dynamic throughout the life span. The personal experiences (3.) that a person has with illnesses are also important for individual knowledge. Individuals can also be differently motivated (4.) to receive information and acquire knowledge. The structural context (5.) for access to information also plays a major role. Differences between urban and rural regions should be considered.

Cognitive and mental level can be divided into factual knowledge (6.), experiential knowledge (7.), and disease knowledge (8.). These three “databases” are connected with each other but can be activated to different degrees. In this context, the individual physiological level can be seen as a “Black Box”. Neurocognitive methods developed in neighboring areas of life sciences have not yet been included in the processing of health information but could provide additional data for the analysis of neurocognitive capacity (9.) and emotional effects (10.) via physiological indicators. This would allow measurement and visualization of differences in emotion while processing health-related information. The level of a person’s health literacy is determined by the overall spread of all the aspects described and depends on how and whether health-related information is understood and processed. To date, most studies on the dissemination of health-related information have been based on data from phenomena 1 to 8 (Figure 1), thereby drawing conclusions about changes in individual health literacy. Expanding the empirical basis by using data from 9 and 10 (“Black Box” in Figure 1) could provide a more complete picture. Certain aspects of different information may activate different brain areas [17,18]. These are neuronal networks which differ in activation strength associated with, for example, emotional, rational, abstract, or concrete processes. Since the physiological parameters of emotional-affective changes are sufficiently known, their extent is measurable. Thus, the neuro-cognitive impact of health information can be determined, even if only indirectly, via the emotional accompanying states.

In order to answer the question about the content of this “Black Box” (see Figure 1), the traditional field of public health needs to be expanded and other disciplines and their standardized methods and existing knowledge have to be adapted. Certain areas of neuroscience and neuropsychology could play a decisive role here. Knowledge about neuronal active structures and mechanisms in the processing of health-related information could provide important insights into the amount and speed of processing. Regarding the topic of stress reduction and prevention of negative effects on health, Can et al. [19] have compiled a survey of studies that use neurophysiological measurement methods to classify stress levels. However, some of the sources given are pilot studies and not fully elaborated experiments. Furthermore, the contributions mentioned in Can et al. [19] focus on the technology and not on the underlying public health problem. However, such a methodological transfer is possible, and to give an example from another field, neuromarketing insights have achieved positive results for marketing campaigns [20]. Here, in advertising and sale strategies, certain brain regions that were associated with the memories of potential customers were target-oriented and activated together with those that were relevant to emotions, which seemed to influence purchasing decisions. The question is whether such target-oriented coupling could be transferred to health-related decision-making based on previously received health information. To our knowledge, it is not yet known what indicators of neurophysiological emotional indicators respond to health-related information. In order to close this research gap, basic insights are needed that provide information on how simple health-related information is processed at the neurocognitive level in adults.

New methods are not only used in testing but also in teaching health literacy, such as audio-visual educational material [21] or virtual/augmented reality via smartphones [22] overviewed in Aida et al. [23] In the context of such studies, the use of neuromarketing methods as well as cognitive neuroscience can be seen as new possibilities [21]. Measuring health may refer to the measurement of general health status through general health measures, measures of physical function, pain measures, social health measures, psychological measures, quality of life measures, and specific disease measures. In this article, we provide an overview of the further possibilities for adapting measurement methods to cover research gaps in the field of health-related information processing and to discuss the possible interaction of physiological measures and correlating emotional–affective states that are triggered by dealing with individual health-relevant issues and their specific ways of processing. Therefore, we generate a narrative review of different ways of studying neurocognitive phenomena and physiological data that includes health information.

## 3. Presentation of Possible Methods for Measuring Physiological Variables and Correlating Emotional–Affective States

In the following, we describe possible methods for measuring the physiological correlates of emotional affective changes that could occur parallel to the processing of health-relevant information (see Table 1). An important advantage of such physiological methods is that they can also be used to determine preconscious sensations or fears. Such unconscious attitudes or fears are difficult to determine through a survey or questionnaire study. The following list of possible methods is not intended to be research instructions for methodical novices in this field. The list of methods is only intended to provide initial information in order to identify possible cooperation partners for future research projects. All of these methods require a high level of expertise in design planning, implementation, and statistical analysis of the data. Furthermore, they differ in terms of effort and costs. The assessment of requirements and costs is only intended to make clear whether the effort involved is high or even very high in order to enable an initial cost–benefit calculation.

### 3.1. Measurement of Respiration Rate, Eye Movement, and Reaction Time

During the processing of relevant health information, a test subject may show emotional changes due to psychophysiological reactions which affect respiratory rate as well as intensity and time course of breathing. A simple technique to record changes in the intensity and time course of breathing is to use a hot-wire anemometer, in which a flow sensor (hot wire) is attached below the nose in the breathing airflow. If this wire is slightly heated electrically and its temperature is measured by a sensor, the cooling effect on the measuring wire caused by breathing air can be determined and displayed by an oscilloscope or computer. While processing health related information, psycho–physical tension or relief can be easily detected because they are noticeable in the person’s respiratory process through pauses or changes in intensity. For example, Boiten [24] shows how different emotional states like disgust, sadness, or joy elicited by different movie sequences are reflected in the volume of air, the rhythm, or the course of breathing.

Analyzing eye gaze and eye movement patterns with eye tracking techniques provides reliable data on attention, selective perception, and the time course of visually perceived information [25,26]. How long certain text passages are viewed (foveal fixation duration), which text passages are skipped (saccades), or whether there are backward jumps (regression) can easily be determined. These data would allow further insight in individual effort, efficiency, and attractiveness of acquiring health related information.

The measurement of reaction time, decision time, or response time by keystroke or vocal response time (“voice key”) also provides very reliable results on the perception and processing of information as well as on the availability and retrieval of information [27]. D’Mello et al. [28] show in their overview such a monitoring of educational processes.

### 3.2. Measurement of Heartbeat/Pulse Rate

Physiological parameters of the heartbeat (heart rate, spectral parameters, blood pressure, etc.) can also be recorded and provide indications of psycho–physical changes related to the examination of affective health information [29]. It can be measured using skin electrodes attached to the left side of the chest (electrocardiogram, ECG). The pulse rate can also be measured using optoelectronic sensors worn on the wrist (fitness tracker, pulse watch). The same is possible with a small pulse oximeter at the fingertip, which opto–electronically measures changes in the oxygen saturation of the blood [30]. In contrast to other methods, this technique is less sensitive for language-based experiments. However, when applying these techniques, abruptly occurring strong emotional sensations are also very well-represented in individual cases. For example, it would be possible to measure the stress level in critical peer-to-peer counseling sessions. Slowly building tension may also be detected. The combined measurement of pulse rate and oxygen measurement is therefore also suitable for individual case analyses with stronger stimuli.

### 3.3. Measurement of Cortisol Level

If the perceived stress level in a person caused by health information is to be determined, the cortisol level (“stress hormone”) can be measured. A stress-causing stimulus can be determined after a few seconds on the basis of the cortisol level in the blood and after one to two minutes when measured in saliva. However, due to the small time resolution, the time window for the testing phase has to be at least one to several minutes. In addition, the time windows of the test and the control condition deliver only a single data point each. Accordingly, additional time must be scheduled for a longer series of critical and control items, or a higher number of participants is necessary. This method can be used, for example, to measure the stress level of people in waiting rooms of emergency admissions. It can also be used to measure whether people experience stress when making health-related decisions.

### 3.4. Measurement of Electrodermal Activity (EDA)

It is implicit that processing health information leads to cognitive processes based on neural activity in a person’s brain. Furthermore, this cognitive processing is accompanied by autonomic nervous system correlates of emotions, leading to changes in electrical skin properties. To measure these electrodermal changes, two electrodes are placed on the skin, and a small electrical voltage is applied. By controlling the electrical current through the measuring device, the time course of the skin’s electrical conductivity can be determined, showing the electrodermal activity (EDA). The test subject has to be deprived and calm, usually sitting in a sound-reduced test cabin. Psychological, emotional–affective, and cognitive processes influence the vegetative changes in skin conductivity in such a way that both conscious and non-conscious (subliminal) processing can be detected.

In a similar way, emotional–affective topics of conversation can be detected by measuring the changes in the electrical properties of the skin [31,32]. By using two skin electrodes at palmar sites of the hand, the measurement of electrodermal activity can begin, for example, while the test subject listens to different health-informative statements. As with the other methods mentioned here, EDA measurements do not produce reliable results based on a single item measurement. Random effects are observed too often for this. However, if 10 to 15 matched stimuli within one participant are compared with an equal number of control stimuli, reliable statements can be made. Broadening the experiment to an additional 10 to 30 participants then also allows a generalization of the findings. According to Visser et al. [33], cancer patients forgot 20 to 80% of the information provided during oncological medical consultations. Since Vissen et al. [33] assumed emotional stress to be the cause of this forgetting, they measured psychophysiological arousal using skin conductance level and heart rate. In the first study, Vissen et al. [33] were unable to show any correlation between psychophysiological arousal, self-reported emotional stress, and memory limitation regarding cancer-related information. Further differentiated studies could close these research gaps. The use of these methods to measure health literacy-associated aspects such as engagement is discussed by D’Mello et al. [28].

### 3.5. Measurement of Electrical Muscle Activity: EMG

Cognitive processing is often accompanied by changes in muscle tension of the mimic face muscles, leading to minimal facial movement. Even if there is no observable facial movement, the smallest electrical activity can be detected by electromyography (EMG). Dealing with affective information is associated with varying muscle tensions, which can be recorded online in their temporal course by EMG electrodes on the facial skin with a temporal resolution in the tenths of a second range [34]. The amplified electrode signal is computer evaluated, leading to stimulus-related EMG amplitudes and time courses. Using electromyography of facial movement, the individual stress level, cognitive arousal, and emotional reaction corresponding to health-related information can be identified over time. A combination of EDA and EMG techniques can be used, for example, to analyze the reading of written or computer-presented health information materials. For example, the extent of emotional engagement in health-related topics can be determined by facial mimic and electrodermal changes and used as a physiological parameter for the reaction to health topics. In the context of general education, attempts are even being made to record such parameters automatically and online [28].

### 3.6. Functional Magnetic Resonance Imaging (fMRI)

The use of brain imaging techniques such as functional magnetic resonance imaging (fMRI) allows a direct view into the working brain during cognitive processes by applying a strong magnetic field and weak electromagnetic waves in a brain scan [35,36]. However, the procedure requires a strictly controlled laboratory situation and is associated with a very high level of effort. During the MRI measurement, the data for the structural anatomical image of the brain is recorded first. After a few minutes, the anatomical data is available, which allows a fine-grained image of the anatomical conditions of the brain in any sectional plane. Following this anatomical measurement, the functional part of the MRI shows the actual brain activity for the given cognitive task. Next, the oxygen level of the red blood cells in the brain is determined, leading to blood oxygen level-dependent imaging. Based on selective oxygen consumption, the activity of the nearby neurons is deduced with a latency of minimally two seconds. Two experimental conditions are always tested, which differ from each other in only one condition: a critical condition (e.g., relevant health information) and a control condition (e.g., neutral health information). By subtracting the activation pattern of the control condition from the test condition, the difference in the activation pattern appears, which shows, in the example above, the information-specific brain activity that is attributable to the critical health information because the brain activities present in both conditions are eliminated by the subtraction. Even though the fMRI method allows a spatially exact view of brain activity distribution, the measurement of activity patterns associated to processing of certain health-related information is almost unexplored.

### 3.7. Electroencephalography (EEG)

Electroencephalography (EEG) allows for non-invasive investigation of the electrical activity of neurons via skin electrodes on a person’s head [37]. While brain imaging methods (such as fMRI) provide information on neuron activity based on oxygen consumption, electrophysiological methods like EEG allow the direct measurement of electrical brain activity with no latency and a temporal resolution in the range of milliseconds, for example, in the perception of factual knowledge and the processing of new information. Electroencephalography (EEG) involves attaching electrodes to the scalp, and the derived signals are amplified and displayed. The EEG is the summed electrical activity of several thousands to millions of neurons underlying each electrode, which is recorded as electrical sum potential. A single stimulus event, such as listening to a single event of health- related information, does not elicit an effect immediately visible in the EEG signature. The data must be averaged over at least 10 to 20 matched stimuli against a control condition in order to detect the desired change in electrical activity from the high EEG background noise using a technique of analyzing the so-called event-related potential (ERP analysis) [38]. However, mobile EEG recording using the P300 component of the ERP as a possible measurement tool for mental health monitoring is shown by Nooner and Kerupetski [39].

## 4. Discussion

It has long been argued that the fast-growing field of health literacy needs more appropriate measurement tools to develop the underpinning theory [40,41]. An overview of the development of objective and subjective measurement tools for health literacy assessment is provided by Altin et al. [42] and Liu et al. [43]. The test procedures used mostly measure literacy skills, but measurement tools have also been developed that test real objects and skills [44]. Previous results of interventions do not show the desired results in terms of understanding and application of modified health-related information. There seems to be a kind of “Black Box” (see Figure 1) that represents the connection between health-related information and its understanding and handling. Applying neuroscience methods to this topic could contribute to clarification. It might be assumed that certain health information causes different and detectable emotional reactions in individuals. Health information would then be processed differently than factual/concrete information, and the processes of administration would be based on many other aspects.

The methods presented here differ not only with regard to necessary requirements and cost (see Table 1). We have evaluated low requirements to be if the method can be performed without much effort. Low effort means that the method can be applied in 2–3 days, and no special laboratory is necessary, so that it is possible to perform the measurement in a clinic or at the test subject’s home. Medium means that a learning period of several days is necessary, the implementation requires sensitive handling, and no special laboratory is necessary. We rated high and very high if the training period lasts several weeks or months, and handling is necessary in a demanding laboratory situation. A special laboratory is required to apply the method, and the person must be very familiar with the measuring instrument. In both cases, it makes sense to cooperate with an established laboratory for a successful research project. Therefore, it should be considered that some methods severely restrict the mobility of the test subjects (e.g., fMRI, EEG, eye tracking). 

The costs range from low (1000–5000 €), medium (5000–30,000 €), high (30,000–150,000 €), to very high (more than 1.5 Mio €). Furthermore, there are strong differences in the latency between the test situation and the evaluation of the results. With online latency, the test subject’s reaction can be seen directly after the stimulus. In the case of measuring the cortisol level and the EDA, the reaction can only be seen after a few minutes. In any case, a data analysis is necessary to check statistical significance. With offline latency, no reaction of the test subject can be detected in the data directly after the stimulus. Only a complex offline evaluation shows possible differences between the tested conditions. If, for example, the immediate effect of health-related information or a specific moment in time of viewing health-related images is to be investigated, EEG, EDA, fMRI, pulse rate, respiratory time, and eye tracking can be used as examination methods. If, on the other hand, the more comprehensive effect of a longer exposition to health-related aspects is to be recorded over a period of one or more minutes, EMG, breath measurement, heartbeat measurement, cortisol determination, and, if necessary, fMRI can be used as examination methods.

Furthermore, of particular importance for choosing among the respective techniques used to measure affective–emotional effects or attention reactions are the number of stimulus-type repetitions required for such an experiment.

The ongoing development of powerful analytical techniques and the further discovery of novel examination procedures show that the methods and examination procedures of cognitive neuroscience have great potential for research into the processing of health-related information. A high effort and a necessary laboratory situation as well as the demands of the examination parameters may sometimes complicate the examination of highly complex stimuli in public health. In addition, in other disciplines such as perceptual psychology or neurolinguistics, initially very simple but then increasingly complex questions have been successfully dealt with. This development can also be assumed for the evidence-based investigation of health information. At present, at least visually or audibly mediated basic emotions can be reliably recorded easily. Real cognitive effects, such as learning processes induced by health information, changes in self-image, changes in attitudes, etc., are unlikely to be recorded in the very near future using neurophysiological techniques but rather continue to be recorded using sociological and psychological methods.

Dealing with health-related information is not only influenced by causal characteristics, but social-emotional factors also play a major role [13]. To measure the interaction of physiological and correlating emotional–affective states triggered by the treatment of individual health-related issues only pre-existing conditions, family burden, or age-related health issues are relevant. The processing of health-relevant information is accompanied by emotional changes if one clearly belong to the target group [45]. If, for example, people whose close family members have already been diagnosed with colorectal cancer are informed about a colonoscopy, it could be comparatively easy to detect an emotional–affective change in these people. If no change in the emotional–affective area could be measured, this could imply that the person does not perceive him/herself as a target group, already possesses a surplus of corresponding information, or has not adequately processed and understood the information.

For measuring the neuronal response to health issues, methods such as fMRI or EEG can be used even though only small effects, characterized by neuronal activity differences, are expected. For this reason, a sufficiently large group must be examined in order to be able to make significant statements. Conversely, it would therefore not be possible to examine and assess the individual extent of successful processing of health-relevant information in a single person. On the other hand, recording the accompanying affective emotional processes using physiological measurements in the processing of health information provides direct access to the physiological reactions and thus the recording of preconscious effects [45]. Individual case studies would also be possible.

It is important to generate findings that reveal patterns in specific target groups and conditions. It might be possible to tailor information to specific conditions and thus make it easier for individuals to deal with the information. Knowledge about the recording of accompanying affective emotional processes using physiological measures in the processing of health-related information could be a profitable component in the conception of targeted interventions that focus on strengthening health literacy and the associated informed and self-determined decision-making.

## 5. Conclusions

Neurophysiological techniques could contribute to research questions on physiological correlates of emotional–affective reactions in the processing of health-relevant information. These findings could be used to develop adapted interventions that design health-related information for different target groups. It could be possible that people with chronic diseases react differently to information in terms of emotional affectivity than close relatives of patients with another disease. The neurophysiological investigation of the processing of health-related information could therefore contribute to the design of information for specific target groups and thus strengthen knowledge and health literacy, particularly to be able to determine preconscious feelings or fears, which are difficult to determine through a survey or questionnaire study.

Studies indicate that the personal ability to assess the reliability of health-related information is relevant, and this can not only be explained by social phenomena, but also measured by physiological correlates. What part emotional states and contexts play in this must be clarified in future studies.

## 6. Limitations

This narrative method overview is lacking in some respects. For example, research using these methods must also consider that social, economic, or in this case, other physiological or psychological factors may have an impact on how health-related information is handled. The methods mentioned here require a certain amount of expertise. Rather, this is a call to engage interdisciplinary partners, to build networks, and to use the disciplines together for the application of the methods.

## Figures and Tables

**Table 1:** Examples of techniques for indicating physiological correlates of the emotional affective changes caused by processing individual health-relevant information.

Measurement	Sensor	Physiological Indicator	Single Event	Requirements	Costs	Latency
Response time, decision time	button box, computer	time of perception, processing, and motoric response	yes	low	low	on-/offline
Eye tracking	infrared eye cameras	eye gaze, fixations, and saccades	yes	medium	medium	offline
Respiratory time course	hot wire sensor, oscilloscope	neurocognitive changes of breathing rate and intensity	yes	low	low	online
Changes in heartbeat/pulse rate	ECG electrodes, amplifier/pulse oximetry sensor	i.a. stress level	possible	low	low	online
Cortisol level	blood sample or saliva testing device	i.a. stress level	possible	low	low	on-/offline ≈ 20 min
Electromyography (EMG)	EMG electrodes, amplifier	neurocognitive changes to muscle tension	yes	low	low	offline
Electrodermal activity (EDA)	EDA electrodes, amplifier	emotionally induced changes to electrical skin resistance	possible	low	low	on-/offline ≈ 2 s
functional magnetic resonance imaging (fMRI)	MRI scanner	brain activity in terms of metabolic demands	no, limited	very high	very high	offline
Electroencephalography (EEG)	EEG electrodes, amplifier	electrical activity of brain areas	no	high	high	offline
